# Studies on Monitoring and Tracking Genetic Resources: An Executive Summary

**DOI:** 10.4056/sigs.1491

**Published:** 2009-07-20

**Authors:** George M. Garrity, Lorraine M. Thompson, David W. Ussery, Norman Paskin, Dwight Baker, Philippe Desmeth, D.E. Schindel, P.S. Ong

**Affiliations:** 1 Department of Microbiology & Molecular Genetics; Michigan State University, East Lansing, Michigan, USA; 2 Pair of Docs Consulting, Saline, Michigan, USA; 3 Department of Systems Biology, Technical University of Denmark, Lyngby, Denmark; 4 Tertius, ltd, Oxford, UK; 5 Acton, Massachusetts, USA; 6 BCCM-Belgian Coordinated Collections of Micro-organisms, Belgian Science Policy Office, Brussels, Belgium; 7 National Museum of Natural History, Smithsonian Institution, Washington, DC, USA; 8 Institute of Biology, University of the Philippines, Quezon City, Philippines

## Abstract

The principles underlying fair and equitable sharing of benefits derived from the utilization of genetic resources are set out in Article 15 of the UN Convention on Biological Diversity, which stipulate that access to genetic resources is subject to the prior informed consent of the country where such resources are located and to mutually agreed terms regarding the sharing of benefits that could be derived from such access. One issue of particular concern for provider countries is how to monitor and track genetic resources once they have left the provider country and enter into use in a variety of forms. This report was commissioned to provide a detailed review of advances in DNA sequencing technologies, as those methods apply to identification of genetic resources, and the use of globally unique persistent identifiers for persistently linking to data and other forms of digital documentation that is linked to individual genetic resources. While the report was written for an audience with a mixture of technical, legal, and policy backgrounds it is relevant to the genomics community as it is an example of downstream application of genomics information.

## Background

There is a natural tendency for practitioners within any given field to focus intently inward on subjects of common interest, and to communicate, oftentimes to the exclusion of all others about those things that are of common interest. In fields that are rapidly evolving, such as genomics, this makes it difficult for those outside the community to appreciate the ramifications that current developments might have on discussions on larger issues. Likewise, those engaged in topics that are peripheral may be equally unaware of how new technologies may affect the interpretation and social policies, regulations, or laws at the national, regional, or international level. Recently, we were invited to contribute our shared views on such a topic, as co-authors of a white paper for the Secretariat of the UN Convention on Biological Diversity (CBD) [1,2]. The CBD was adopted in 1992 and entered into force in 1993. At present, there are 192 Parties (nations) to the convention.

The three principal objectives of the CBD are: conservation of biological resources, sustainable use of its components, and fair and equitable sharing of benefits arising out of their utilization. The principles underlying fair and equitable sharing of benefits deriving from the utilization of genetic resources are set out in Article 15 of the CBD, which stipulates that access to genetic resources is subject to the prior informed consent of the country where such resources are located and to mutually agreed terms regarding the sharing of benefits that could be derived from such access.

Further to a call for action by the World Summit on Sustainable Development in 2002, the Conference of the Parties to the CBD mandated a subsidiary body to negotiate an international regime on access and benefit-sharing in order to further enable Article 15 and other relevant provisions of the CBD [3,4]. At COP 9, in May 2008, it was agreed that negotiation of the international regime should be completed by COP 10 in October 2010.

One issue raised during the negotiation process, which is of particular concern for provider countries, is the monitoring and tracking of genetic resources; that is to say what happens to those resources once they have left the provider country and enter into use in a variety of forms. Previously, the issue of monitoring and tracking had been considered in discussions about an internationally recognized certificate of origin/source/legal provenance. An expert group was established by COP to further examine this potential tool or instrument that could assist to track or trace genetic resources.

At COP 9, in relation to the issue of monitoring and tracking, the Parties requested that two studies be carried out to inform the negotiation process (decision IX/12., paragraph 13):

- Recent developments in methods to identify genetic resources directly based on DNA sequences;
- To identify the different possible ways of tracking and monitoring genetic resources through the use of persistent global unique identifiers, including the practicality, feasibility, costs and benefits of the different options;

Against this background, we were commissioned to provide a detailed report that could inform the negotiation process on these issues. The studies we prepared were aimed at the negotiators of the international regime, and are written for an audience with a mixture of technical, legal, and policy backgrounds. However, the topics we discuss are also relevant to the genomics community and we welcome any comments the readers of SIGS might like to share.

## Introduction

Technological innovations, in areas such as DNA sequencing and information technology are characterized by exponential development rates and lead to results that are typically unanticipated when first introduced. Three examples demonstrate this clearly. In 1995 it took Fleischmann *et al*. thirteen months to sequence the complete genome of Haemophilus influenzae at a cost of approximately fifty cents per base pair [5]. Today a bacterial genome can be sequenced in less than a day for pennies per base pair and the possibility of sequencing a complete bacterial genome in a few hours for under $1000 looms in the near future [6]. In 1983 TCP/IP, the underlying protocol of the Internet became operational [7]. As of June 30, 2008, 1.463 billion people use the Internet according to Internet World Stats with the greatest growth in usage between 2000-2008 occurring in Africa (1,031.2%), Latin America/Caribbean (669.3%) and Asia (406.1%) [8]. On August 6, 1991, the European Organization for Nuclear Research (CERN) publicly announced the new World Wide Web project [9]. Eighteen years later the Indexed Web contains at least 25.9 billion pages) [10]. According to UN statistics, 64% of all mobile phone users can now be found in the developing world. With a compound annual growth rate of 49.3% over the last seven years, Africa has become a key market for global telecom operators; and it is expected that this market will continue to grow faster than any other region over the next three to five years [11,12]. In parts of Africa, health teams are synchronizing their mobile devices and collecting data from rural clinics to provide better health care [13]. Clearly the digital divide that once existed is closing rapidly and databases and other digital resources are accessible today to anyone, anywhere, with an internet connection and a browser on a computer, a handheld device, or even a cell phone.

It is in this environment of rapid technological innovations and global information access in which the CBD must work to ensure the *sustainable use* of biodiversity as a means to justify and underwrite its preservation. As part of this effort an international regime (IR) on accessing genetic resources and sharing benefits derived from their utilization is currently being negotiated by the Conference of Parties of the CBD [3]. The purpose of this paper is to assist the COP in thesenegotiations by providing a detailed examination of the following technical issues:

- Recent developments in methods to identify genetic resources directly based on DNA sequences;
- Identification of different possible ways of tracking and monitoring genetic resources through the use of persistent global unique identifiers (GUIDs), including the practicality, feasibility, costs and benefits of the different options.

This paper presents the overall summary from a more extensive Access and Benefit Sharing report made for the United Nations.

## Genetic Resources

Genetic resources are used worldwide by many different industries, academic institutions, and environmental organizations to achieve various goals, ranging from developing new commercial products and processes to exploring new research avenues for cataloging and preserving biotic specimens arising from biodiversity inventories. In Article 2 of the CBD, genetic resources are defined as “genetic material of actual or potential value” and are further defined as “any material of plant, animal, microbial or other origin containing functional units of heredity.” The value of these resources need not be exclusively genetic material. It may also be derived information, such as functional or regulatory pathways, structural polymers or biological functions of an organism that are encoded for by the genetic material, including metabolic products that have some practical applications (*e.g.*, low molecular weight organic acids; anti-microbial agents, such as antibiotics, and other biopharmaceuticals, flavors and fragrances, enzymes for industrial applications).

## Provenance, tracking and terms of use

Currently, the use of, and access to, specified genetic resources are governed by contractual agreements between the providers and users of those resources. For the purpose of this study it is assumed that such agreements are in compliance with all the relevant existing legal and other instruments at national, regional, and international levels relating to ABS. Contractual negotiations that follow the voluntary Bonn Guidelines result in a set of accompanying documents that explicitly detail the terms of any agreement including prior informed consent (PIC) and material transfer agreements (MTAs) and possibly Mutually Agreed Terms (MATs) and Certificates of Origin (CoO). Such documents by themselves do not provide a means by which a specified genetic resource(s) can be singled out and tracked, but do establish an important part of the baseline information that must be collected and made accessible to various parties to the agreement. These agreements also establish the conditions for access to both the resources and information over time and should also specify what types of information are required to follow along with any genetic resource and any real or abstract derived products, either for fixed periods of time or in perpetuity. With this minimal information in hand, it becomes possible to devise reasonable and extensible models to track each genetic resource as it moves from its point of origin through one or more user organizations for a variety of purposes.

It should be understood that a large-scale tracking system that meets the needs of the IR does not yet exist. Smaller-scale implementations do, however; and have features that are desirable in the anticipated tracking system for genetic resources. These are discussed in detail in part II of the extended published report [2]. We have drawn from prior experience with those smaller scale systems to gain useful insights into the requirements of a robust, reliable, and trustworthy tracking system that could accommodate the needs of a diverse end-user community working in pure and applied research, international trade, regulation, and enforcement. It is important to stress that development of a complete tracking system for genetic resources must consider non-technical issues as well, including realistic policies that address complex social, business, and scientific requirements. This will ensure widespread acceptance and usage. It is not uncommon for technically sound information systems to fail because user needs were not met or the system rigidly modeled practices that became obsolete because of changes in technologies external to the system, but critical to the organizational goals, that were not anticipated or could not be incorporated into the system. This is particularly true in the life sciences and is discussed in part III of the extended report [2].

## Redefining genetic resources

Whereas whole organisms or parts of organisms were once the subject of study and trade, contemporary biology has expanded its focus to incorporate molecular and informatics methods (*in silico*). These newer methods allow us to describe living systems not only on the basis of readily observable traits, but also upon their genetic potential based on a direct analysis of selected portions of the genome or the entire genome. As a result, genetic resources are now being used in various forms ranging from extracted DNA (including from mixed populations in metagenomic studies) to various types of sequence data that are stored in public and private databases. These derived genetic resources are readily copied, mobile, and readily accessible to a global audience and can be used for a variety of purposes (*e.g.*, expression in heterologous hosts, engineered chimeric pathways, synthetic life forms) that may have not been intended or anticipated in original agreements.

Therefore, it can be argued that rights and obligations under the IR may extend to the exploitation of genetic resources, regardless of how those resources are constituted. Although a discussion of the merits of such thinking is beyond the scope of our charge, we believe it prudent to consider the consequences. Under such an interpretation, a system for tracking genetic resources would have to provide a means for providers to track the uses of the data and information derived from their genetic resources. The task of tracking successive uses of such information, although complex, is theoretically feasible and would require the crafting of appropriate metadata, careful utilization and implementation of a persistent identifier system, and development of custom tracking applications (See [14] for an example). However, it should also be understood that such a system would have to accurately reflect our current and future knowledge of biology. The vast majority of gene sequences is ubiquitous in nature and oftentimes occurs in distribution patterns that do not necessarily conform to national boundaries. It should also be understood that current technology allows the rapid synthesis and evolution of genes and pathways *in vitro* and *in silico*. Therefore, apparent misuse of a resource by a user or third party may not be actual misuse. Rather, it may be an instance of coincidental use of a like resource obtained independently. It is with these points in mind, that we offer the Secretariat and the COP our observations and recommendations on the agreed upon topics.

## Single gene identification methods

The rapid development of molecular technologies that enables characterization of organisms at a genetic level has opened new possibilities in species identification. In 1977 Woese and Fox produced the first phylogenetic classification of prokaryotes^1^ based on the comparison of the nucleotide sequence of the 16S rRNA gene [15]. This gene is universally distributed, highly conserved, evolves very slowly, and plays a key structural role in the ribosome, which in turn is part of the cellular machinery involved in protein synthesis. All life forms, as we know them, possess ribosomes, so according to the early proposals of Pauling and Zukerkandel, the sequence of this molecule could serve as a molecular chronometer, by which the evolution of different species could be traced [16].

Woese's work revealed that bacteria and archaea formed two deep and very distinct evolutionary lineages. The third lineage, based on this model of evolution, encompasses the eukaryotes (the plants and animals), which characteristically posses a membrane enclosed nucleus and organalles (including the mitochondria and chloroplasts). Eukaryotes possess ribosomes, which in turn contain an 18S rRNA. The eukaryotic 18S rRNA gene shares many homologous regions with the prokaryotic 16S rRNA gene. Thus, it is possible to make meaningful comparisons of all species based on the sequence of this gene. Since the sequence of the 16S rRNA gene is approximately 1540 nucleotides in length, there is sufficent information content to allow for very far reaching comparisons.

Woese's discovery has led to a radically different understanding of the evolutionary history of all life, which is generally well accepted and has led to the abandonment of alternative models of classification (e.g., Whittaker's five kingdoms). 16S rRNA Sequence analysis has become the principal method by which bacteria and archeae are now classified. In the past two decades, thousands of new taxa have been described based on this method, along with numerous taxonomic rearrangements. Concurrent improvements in sequenceing methodologies have greatly accel-erated this process. Today, 16S rRNA sequence data is routinely used to presumptively identify bacteria and archaea to the genus level and to deduce community composition in environmental surveys and in metagenomic analyses. These efforts are well supported by publicly available tools and highly curated data sets of aligned 16S rRNA [17-19].

But it is now well understood that a single gene may not be adequate to yield an accurate identification to the species or subspecies level and additional gene sequences along with other data may be required. Confounding issues include non-uniform distribution of sequence dissim-ilarity among different taxa and instances in which multiple copies of the 16S rRNA gene may be present in the same organism that differ by more that 5% sequence dissimilarity. This can lead to different presumptive identifications for the same individual, depending on which 16S rRNA gene is analyzed. We also understand that numerous instances of misidentification and taxonomic synonomies have accumulated prior to the widespread adoption of these methods and that discrepancies between names and correct classification remain to be resolve. In such instances, molecular evidence needs to be used to support taxonomic revision rather than attempting to force-fit earlier concepts into a classification based on reproducible molecular and genomic evidence.

These observations are relevant to the development of a tracking system for genetic resources because taxonomic names are commonly used in the scientific, technical, and medical literature as well as in numerous laws and regulations governing commerce, agriculture, public safety, and public health. But taxonomic names are not suitable for use as as they are not unique, not persistent, and do not exist in a one-to-one relationship with the abstract or concrete objects they identify.

Analogous developments are currently underway in the fields of botany and zoology. Sequence based methods have been applied on a limited basis to various species of eukaryotes for many years. However, it was not until recently that the community began to accept the possibility that a single gene could be used for identification of eukaryotes. This approach is now being applied in a highly coordinated fashion to build useful resources to identify plants, animals, fungi, protists, and other distinct eukaryotic lineages. Consensus is beginning to emerge on a small number of preferred target genes, of which a partial sequence of the mitochondrial cytochrome *c* oxidase subunit I gene is preferred. This highly coordinated effort is much more recent than the corresponding activities in microbiology, and championed by the Consortium for the BarCode of Life (CBoL) program [20].

## Implications of genome sequencing

In part III of the extended report, we provide an in-depth review of next generation sequencing (NGS) technologies [2]. Because of the rapid pace at which these technologies are evolving this section should be viewed as a set of “snapshots” of the current state of the art, and a harbinger of the future of DNA based identification methods. We discuss methods that are currently in use; those that have just recently become available on the market, (near-future NGS methods); and those that are still under development. These NGS sequencing technologies enable the rapid evaluation of specific regions of the genome of a biological entity to determine to which genus, species, or strain it belongs (*e.g.,* the *16S* rRNA gene for taxonomic purposes for bacteria; the use of cytochrome *c* oxidase subunit I (*cox1*) for eukaryotes).

Fueled by innovations in high-throughput DNA sequencing, high-performance computing, and bioinformatics, the rate of genomic discovery has grown exponentially. To date, there are more than 500 complete genome sequences and more than 4000 ongoing genome and metagenome sequencing projects covering species ranging from bacteria to yeast to higher eukaryotes. The results that stream forth from these studies are constantly refining and reshaping our understanding of biological systems. As part of the funding requirement of various governmental and non-governmental agencies, the vast majority of these sequences are made publicly available from the INSDC databases after brief embargo periods during which time the funding recipients may publish their results [21]. Typically, after one year, the sequence data is open to anyone wanting to publish their own findings or mine those data for other purposes.

All indications are that future genome-based technologies will be “smaller, cheaper, faster”. This will make genome-enabled detection tools available to a wide audience in both developed and developing nations. Clearly, very low cost sequencing technology along with sophisticated bioinformatics tools will soon be available to presumptively identify a genetic resource, with a high degree of accuracy and reliability, at the point of need.

## Tracking genetic resources

The concept of identification is central to the goals of the CBD ABS regime, which rests on the fundamental principle that a user is legally obliged to share in the benefits obtained through the use of a particular genetic resource with the provider. Identification is one of the first steps in tracking an item over time. Under some circumstances, identification to the family, genus, or species level may be adequate and identification methods based on a single gene may be appropriate (e.g., biotic inventories, wild-life management, ecological studies). However, there is ample evidence based on over half a century of natural product screening and supporting genomic data that such approaches may be inadequate if the trait of interest occurs in subpopulations within a species or is widely distributed across taxonomic boundaries as a result of horizontal gene exchange. A useful tracking system must accurately reflect current knowledge and readily incorporate new knowledge via continuous feedback over a long time frame as transactions involving genetic resources may be long lived (>20 yrs).

The number of items to be identified and tracked within the anticipated system is a challenge and the extent of the task will depend largely on the legally required “granularity” of the identification. Although there is a tendency to view this as a taxonomic problem and the anticipated tracking system as a taxonomic resource, it is decidedly distinct. What is required is a mechanism to track the fate of multiple genetic resources as each is transferred from one party to another and various abstract and concrete products are generated along the way. In some cases the product may be useful for taxonomic purposes and in other cases taxonomic information may be useful for predictive purposes, but in most cases taxonomic information would be ancillary. Systems of such design are challenging as they are open-ended and must work with data of varying granularity. The point is not to define all the types of data *a priori*, but to define lightweight metadata models that define genetic resources and allow them to be permanently bound other to varying amounts and types of information that accumulate about that genetic resource over time. Inherent in such designs are links established through aggregates of foreign keys that may exist within a single system or on a remote systems accessible via the internet.

## Persistent identifiers

In their simplest form, persistent identifiers are nothing more than unique labels that are assigned to objects in a one-to-one relationship. Such identifiers are well understood in computing systems and we present examples of identifiers as used in a large-scale laboratory information management system (LIMS) in Part II of the extended report [2]. When used in the context of the internet, the concept of persistent identification is frequently coupled with the concept of actionability, implying that the PID is persistently linked to a specific object and when actuated, will always return the same response to the end-user (typically a hyperlink to a specific web page or other form of digital content). In this context PIDs differ from URLs, which are used to create hyperlinks and provide the internet address of where a given object is stored. As the storage location is not persistent, some "behind-the-scenes" mapping of object identifiers to object locations is required (resolution). This topic is covered in more detail in part IV of the extended report [2].

Persistent identifiers are a powerful enabling technology that provides a way to efficiently cope with chronic problems such as broken links and the general difficulty of reliable and reproducible information retrieval on the Internet. For example, PIDs associated with published articles allow rapid and accurate tracking of written works. PIDs are also in use within the life sciences such as the INSDC identifiers (*e.g.*, sequence accession numbers used at GenBank, EMBL, and DNA Database of Japan) [22-24]. However, these are largely institution specific, *i.e.*, used only within the institutions for which they were created, or are controlled by those organizations, such as the PubMed ID, issued by the National Library of Medicine.

Six PID schemes currently used across different domains and by a number of different organizations are reviewed and include: Uniform Resource Name (URN [25]:); Persistent Uniform Resource Locator (PURL [14]:); Archival Resource Key (ARK [26]); Life Science Identifiers (LSID [27]); Handle System (Handle [28]); Digital Object Identifier System (DOI [29]). This review also addresses the questions that need to be answered when an organization is assessing the need to incorporate a PID scheme into its data management plan.

Each of these identifiers is used in well-defined settings in which the data and metadata models of the underlying repositories were established *a priori*. The identifiers serve as a means of directly accessing a specific record or other form of digital content or the associated metadata. If the identifier is actionable, then it is possible to retrieve the linked object using the familiar interface of a web-browser. However, with the use of web services that provide structured access to the content of interest automatically (*e.g*. from a database or application on a handheld device using embedded PIDs), similar results can be achieved where an interactive interface is not suitable.

An effective and durable PID scheme requires ongoing maintenance and therefore ongoing resources. While some tasks can be automated, responsibility for this ongoing task must be assigned to an agency, program, or office, or to a trusted third-party who can guarantee reliability and virtually constant up-time to meet the needs of various end-user communities. In the case of integrating a persistent identifier scheme within the ABS process, the use of a trusted third party with the appropriate expertise and resources is probably the best option, especially if that third party is already engaged in such activity for other purposes.

The selection of an appropriate PID for the CBD ABS and related activities will be critical for its broad utility and community acceptance. However, it does not obviate the importance of carefully defining precisely what the identifiers refer to, and what will be returned by queries of various types. It is possible to develop a range of PID services that could, for instance, provide a direct link to digital and paper copies of entire documents, such as PICs, MTAs, CoOs, and other relevant agreements or permit tracking of genetic resources or parts of genetic resources in a future proof method, or do so on-the-fly. It could also be possible to track the transfer of materials and the corresponding agreements to third parties in a manner that is consistent with the rights and obligations of all parties to the initial agreement or to subsequent agreements. Similarly, the ability to track these genetic resources into the STM, general interest and patent literature is technically feasible.

Services such as these could be facilitated through the use of a trusted third party acting as a clearinghouse for registering ABS-related events (*e.g.,* PIC, MTAs, CoO, and other relevant agreements) according to a set of well-understood business rules. With such a clearinghouse in place, it becomes possible to traverse a series of transactions backward and forward in time, even in instances where some ambiguity may exist. By drawing on highly interconnected information, it is possible to follow events, and to accurately recreate those events, when adequate documentation is available. Such a system would be useful for monitoring the use of genetic resources, especially since there will be instances in which long periods of time may exist between the time PICs, MTAs, and CoO are executed and some commercial or non-commercial product results. With the selection of the appropriate PID system, a system of this design could support human and machine queries and facilitate the retrieval of all relevant documents from public and private databases, including the STM literature, patent, and regulatory databases. This is discussed in more depth in part IV (CBD/ABS services) [2].

## Conclusions and recommendations

Reduction to practice will require a commitment of interested parties from different sectors (*e.g*., government, industries, botanical gardens, museums, academia, etc) to define standards for the key documents that are instrumental to implementing the ABS. Business rules and policies also need to be established in concrete terms so that useful prototypes can be built and assumptions (technical, legal, and social) tested and refined. In part V of the extended report we offer the Secretariat and COP five broad recommendations along with our reasoning [2]. In summary, these are:

1. Promptly establish the minimum information that must be contained in all relevant documents that are required for compliance with the IR (PIC, MTA, MAT, CoO). Stipulate which documents are mandatory and which are optional.
2. Adopt a well-developed and widely used Persistent Identifier PID system (*e.g*. DOI) that leverages an existing infrastructure and derives support from multiple sources rather than developing a new system or adopting one that is untested in commercial applications.
3. Carefully consider the current and future needs of genetic resource providers and users as the concept of resource tracking is deliberated. Biological and functional diversity of genetic resources are decidedly distinct. The system, including its human resource component, must be able to accommodate both with priority given to the latter as functional diversity is what leads to practical utility.
4. Deploy light-weight applications that use browser technology for interactive use and publish well documented application program interfaces to support other web service. Develop strong policies governing access and use of the resource to avoid data abuse.
5. Deploy one or several prototype tracking systems to validate underlying concepts and refine critical elements that will be needed in a fully operational system. During the developmental phase address erroneous preconceptions and focus on making the system as transparent as possible.

## Notes

^1^The term prokaryote is a contentious but commonly used name to group bacteria and archaea together based on their absence of a nucleus; a feature found in all eukaryotes
